# When designing vaccines, consider the starting material: the human B cell repertoire

**DOI:** 10.1016/j.coi.2018.08.002

**Published:** 2018-08

**Authors:** Colin Havenar-Daughton, Robert K. Abbott, William R. Schief, Shane Crotty

**Affiliations:** 1 Division of Vaccine Discovery, La Jolla Institute for Allergy and Immunology, La Jolla, CA 92037, USA; 2 Center for HIV/AIDS Vaccine Immunology and Immunogen Discovery, The Scripps Research Institute, La Jolla, CA 92037, USA; 3 Department of Immunology and Microbial Science, The Scripps Research Institute, La Jolla, CA 92037, USA; 4 International AIDS Vaccine Initiative Neutralizing Antibody Center, The Scripps Research Institute, La Jolla, CA 92037, USA; 5 The Ragon Institute of Massachusetts General Hospital, Massachusetts Institute of Technology and Harvard University, Cambridge, MA 02139, USA; 6 Division of Infectious Diseases, Department of Medicine, UCSD School of Medicine, La Jolla, CA 92093, USA

## Abstract

Most viral vaccines provide protection from infection through the generation of neutralizing antibodies (nAbs). The repertoire of B cells responding to immunization is the starting material from which nAbs eventually arise. Immunization strategies are increasingly targeting precise B cell specificities to mimic nAbs generated during natural infection, in an effort to maximize the potency of the vaccine-elicited Ab response. An understanding of the human B cell specificities capable of immunogen recognition can aid in immunogen design and inform decision-making for clinical advancement. Here, we review what is known about antigen-specific and epitope-specific naive B cell repertoires in humans and mice, and we consider the challenges for identifying and analyzing antigen-specific naive B cell repertoires. Finally, we provide a framework for further exploration, interpretation, utilization of the B cell repertoire to facilitate vaccine discovery.

## Highlights

• Antigen-specific naive human B cell repertoire analysis as clinical trial prelude.
• Protein-specific and epitope-specific quantitation of B cells before antigen exposure.
• B cell precursor frequency and affinity strongly impact germinal center responses.
• Antigen-specific B cell repertoire study informs immunogen design and prioritization.

## Graphical abstract

**Figure:**
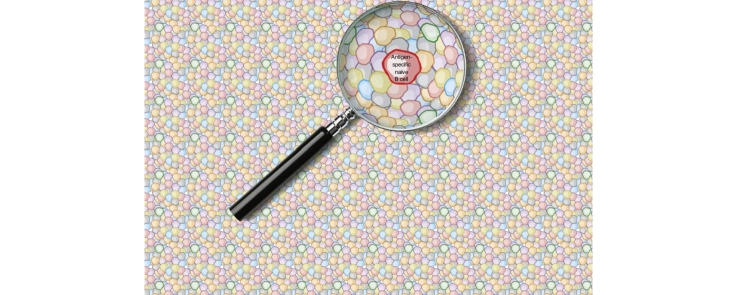

**Current Opinion in Immunology** 2018, **53**:209–216

This review comes from a themed issue on **Vaccines**

Edited by **Patrick C Wilson** and **Florian Krammer**

For a complete overview see the Issue and the Editorial

Available online 3rd September 2018

**https://doi.org/10.1016/j.coi.2018.08.002**

0952-7915/© 2018 The Authors. Published by Elsevier Ltd. This is an open access article under the CC BY license (http://creativecommons.org/licenses/by/4.0/).

Neutralizing antibodies (nAbs) are the protective immune response induced by most human vaccinations against viruses [1]. Difficult to neutralize pathogens, such as HIV and influenza, are major targets for current vaccine efforts. So-called ‘Reverse Vaccinology 2.0’ is an important new approach wherein protective nAbs isolated from infected individuals are used as a road map to design immunogens with the goal of recreating those nAb responses by immunization [2,3]. In the case of HIV, the complexity of the nAb epitopes and plethora of evasion mechanisms employed by the virus [4] may dictate the need for a multi-stage, multi-immunization approach that shepherds B cells past various antigen recognition and affinity maturation impediments to produce broadly neutralizing antibodies (bnAbs) capable of neutralizing a majority of HIV strains [5,6]. A first key challenge to epitope-specific or site-specific vaccine design is that naive B cells with the correct epitope specificity may be both rare and have low affinity for the pathogen. This can result in immunodominant non-protective B cell specificities outcompeting the desired B cells specific for protective epitopes [7, 8, 9, 10]. Help from CD4^+^ T follicular helper (Tfh) cells is likely to be an important factor in the recruitment of rare and/or low affinity B cells but is not the topic of this review; the impact of limited Tfh cell help to B cells is discussed elsewhere [7,11]. B cell receptor (BCR) ‘germline-targeting’ immunization approaches aim to set the B cell response on a track to bnAb generation by narrowly targeting B cells with particular sequence attributes that provide epitope-specificity [12, 13, 14]. The HIV envelope CD4-binding site (CD4bs) targeting immunogen eOD-GT8 is one of the most advanced germline-targeting concepts, with a first-in-class clinical trial scheduled to begin in 2018 [5,8,14, 15, 16, 17, 18, 19, 20]. The goal of eOD-GT8 immunization is to stimulate naive B cells that recognize a modified HIV CD4bs via paratopes comparable to that of the prototypic HIV CD4bs-recognizing bnAb VRC01 (i.e. VRC01-class naive B cells. Compatible paratopes are referred to as c-paratopes here). In transgenic mice engineered to express the inferred germline BCR heavy chain (HC) of VRC01, or related sequences, immunization with eOD-GT8 60-mer nanoparticles was able to prime VRC01-class B cell responses successfully [15,16], demonstrating the validity of the general concept and immunogen design; however, such mice have supraphysiological frequencies of antigen-specific B cells. Central questions remained about whether VRC01-class naive B cells exist in most humans and what the physiological frequencies and affinities were of such naive B cells. This is a general matter of interest for all site-specific and germline-targeting vaccine design efforts.

The naive B cell repertoire in humans is the essential starting material for the generation of antibody responses, and yet little is known about the vast collection of B cell specificities at the epitope-specific or protein-specific scale. Here, antigen-specific naive B cells are defined as B cells in the pre-immune repertoire that are naive (i.e. not antigen-experienced) and have affinity for a specific antigen. For a potential epitope on an immunogen, do epitope-specific B cells within the naive repertoire exist? If so, at what frequency are these B cells found, and are they found in the majority of individuals? What are the binding affinities of those naive B cells for the immunogen? Each of these questions is important for site-specific and germline-targeting vaccine design efforts; however, they have posed challenging technical hurdles. One approach to solve these problems is bioinformatic, examining available BCR repertoire sequence data. While that can be a starting point, key caveats of that approach are the limited HC-LC paired sequence data available for human naive B cells, and limited knowledge of the BCR c-paratope sequence requirements for recognition of a novel immunogen epitope. We developed experimental approaches to address these questions by directly examining the antigen-specific naive B cell repertoire among hundreds of millions of human B cells from multiple donors. Using the eOD-GT6 immunogen [14], we probed the naive human B cell repertoire, specific not only for a single protein, but also for a distinct epitopic site on that protein, the CD4bs. Epitope-specific B cells were isolated, but no VRC01-class B cells were found. These data suggested that eOD-GT6 was unlikely to succeed in a human clinical trial [17]. This helped provide the basis for iterative vaccine design, to develop an improved eOD-GT, eOD-GT8, with improved CD4bs epitope characteristics. Using the eOD-GT8 immunogen, we again probed the naive human B cell repertoire, and in those experiments a robust number of VRC01-class naive B cells were found, demonstrating the design success of eOD-GT8 [17,21]. Conceptually, analysis of the antigen-specific naive human B cell repertoire can be thought of as an *ex vivo* human clinical trial prelude, with the goals of identifying vaccine-specific naive B cells and providing information on whether a given vaccine design is likely to accomplish the goal of targeting specific protective Ab responses. These data can inform iterative vaccine design, as they did for eOD-GT6 to eOD-GT8. This experimental approach can also be one criterion for comparative assessment of different immunogens for advancement along the development pathway to human clinical trials, given the expense and time costs for human clinical trials.

eOD-GT8 was designed to bind to germline-inferred precursor B cells of the VRC01 bnAb lineage and related VRC01-class bnAbs. VRC01-class bnAbs all bind to the HIV Env CD4bs in a distinct structural manner using a BCR encoded by a VH1-2 HC paired with a LC encoding a 5 amino acid (aa) L-CDR3. Thus, we define VRC01-class naive B cells as VH1-2^+^ B cells with a 5aa L-CDR3 and confirmed specificity for the modified HIV Env CD4bs of eOD-GT8. We identified a total of 69 human VRC01-class naive B cells via eOD-GT8 binding [17,21]. The VRC01-class naive B cells had a mean affinity of 3 μm *K*_D_ for eOD-GT8 (range: *K*_D_ = 13 nm to 100 μm). This seemed to be a strong affinity, given that the naive B cells have not undergone somatic hypermutation, but there are very limited examples of affinity measurements for other repertoires of epitope-specific human naive B cells [48] and therefore limited conclusions could be made. This is a knowledge gap in the field. A structure was solved of one representative VRC01-class naive B cell-derived Ab in complex with eOD-GT8, showing that the VRC01-class naive B cell engaged the CD4bs on eOD-GT8 with a paratope and angle of approach comparable to that of bnAb VRC01 on HIV Env. Importantly, VRC01-class naive B cells were identified in the majority of tested donors, and a simple Poisson analysis suggested that the vast majority of humans have VRC01-class naive B cells in their repertoires [17]. The frequency of VRC01-class B cells was ∼1 in 300 000 B cells (Table 1) [17,21]. Each of the parameters described above, determined by direct examination of the human naive B cell repertoire, provided valuable information for pre-clinical and clinical development.

**Table 1:** Known and predicted frequencies of antigen-specific B cells before antigen exposure

Antigen	Epitope	Technique	Frequency	Tissue (species)	Ref
**Class-specific, epitope-specific B cells. Protein antigen**					
eOD-GT8. eOD-GT8^KO^	CD4bs of HIV Env.^a^	Flow cytometry + sequencing + Ab expression	VRC01-class naive B cells 1:300 000	Blood (*Homo sapiens*)	[17,21]

**Epitope-specific B cells. Protein antigen**					
eOD-GT8. eOD-GT8^KO^	CD4bs of HIV Env.^b^	Flow cytometry + sequencing +Ab expression	1:40 000	Blood (*Homo sapiens*)	[17,21]

**Whole protein antigen-specific B cells**					
Phycoerythrin (PE)	Unknown	Flow cytometry	1:5000	Spleen, LN (*Mus musculus*)	[40]
Allophycocyanin (APC)	Unknown	Flow cytometry	1:25 000	Spleen, LN (*Mus musculus*)	[40]
Anthrax PA	Unknown	Flow cytometry	1:8000	Spleen (*Mus musculus*)	[25]
Influenza HA	Unknown	Flow cytometry	1:18 000	Spleen (*Mus musculus*)	[25]
Sheep red blood cells (SRBC)	Unknown	*In situ* hemolysis + limiting dilution cell transfer	1:12 000	Spleen (*Mus musculus*)	[41]

**Hapten specific B cell precursors**					
4-Hydroxy-3-nitrophenyl acetyl (NP) – PE or APC or A647/BSA	Unknown	Flow cytometry	1:4000	Spleen (Mus musculus)	[42]
4-hydroxy-3-nitrophenyl acetyl (NP) – APC	4-Hydroxy-3-nitrophenyl acetyl (NP)	Flow cytometry	1:5000	Spleen (*Mus musculus*)	[43]
2,4,6-Trinitrophenyl (TNP)-hemocyanin	2,4,6-Trinitrophenyl (TNP)-specific	Splenic focusing	1:10 000	Spleen (*Mus musculus*)	[44]
Fluorescein isothiocyanate (FITC) – hemocyanin	Fluorescein isothiocyanate (FITC)-specific	Splenic focusing	1:15 000	Spleen (*Mus musculus*)	[44]
Phosphorylcholine (PC) – hemocyanin	Phosphorylcholine (PC)-specific	Splenic focusing	1:50 000	Spleen (*Mus musculus*)	[45]
*p*-Azophenylarsonate – hemocyanin	*p*-Azophenylarsonate	Splenic focusing	1:66 000	Spleen (*Mus musculus*)	[46]
5-Dimethylaminoapaphthalene-sulfonyl-hemocyanin	5-Dimethyl-aminoapaphthalene-sulfonyl	Splenic focusing	1:10 000	Spleen (*Mus musculus*)	[46]

a Bound eOD-GT8 and not the epitope-mutated eOD-GT8^KO^, the HC was encoded by VH1-2, and the L-CDR3 was 5aa in length.

b Bound eOD-GT8 and not the epitope-mutated eOD-GT8^KO^. No sequence defined restrictions.

Binding of VRC01-class naive B cells was the design goal of eOD-GT8; they can be termed on-target B cells. In addition to these cells, other naive B cells in the human repertoire with the ability to bind eOD-GT8 were identified and organized into three off-target categories based on BCR sequence and epitope specificity (Figure 1). These non-VRC01-class naive B cells likely represent competitor B cells, which may be a problematic aspect of any novel protein design. Off-target B cells for eOD-GT8 include firstly CD4bs-specific VH1-2^+^ naive B cells with non-5aa L-CDR3 lengths, secondly CD4bs-specific non-VH1-2 naive B cells, and thirdly non-CD4bs specific naive B cells. Importantly, direct examination of the human naive B cell repertoire revealed that these off-target B cells were more common that VRC01-class B cells (Table 1) but had lower mean affinities to eOD-GT8 [17,21]. This suggested that in humans immunized with eOD-GT8 60-mer immunogen, VRC01-class naive B cells would have an affinity advantage for activation, germinal center (GC) recruitment, and memory formation, but would be disadvantaged by their rarity in the naive B cell repertoire compared to off-target B cells. These naive B cell affinity and precursor frequency considerations are likely relevant to other engineered protein vaccine designs.

**Figure 1:**
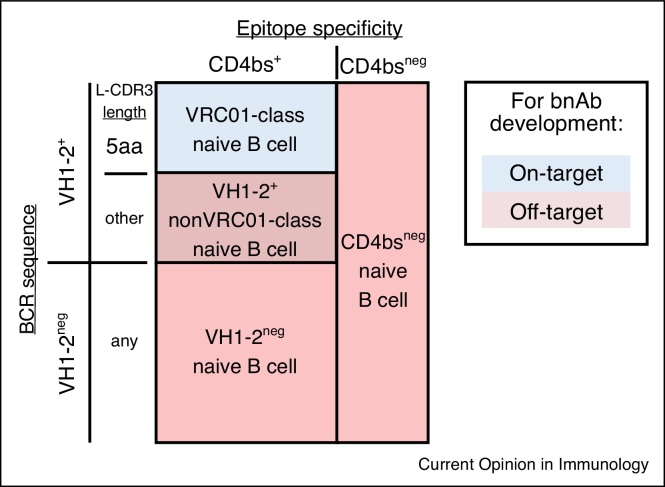
Classification of eOD-GT8 specific naive human B cells by epitope specificity and BCR sequence characteristics. The colored area represents the eOD-GT8 specific naive human B cell repertoire. ‘On-target’ naive B cells (blue) with BCR sequence characteristics (VH1-2^+^ and 5aa L-CDR3 length) and CD4bs epitope specificity are classified as VRC01-class naive B cells.

## The relevance of precursor frequencies

Is precursor frequency a limiting factor for successful B cell responses during vaccination? Is affinity a limiting factor? If so, what, if any, antigen properties can affect successful B cell outcomes? Our understanding of epitope-specific naive B cell repertoire precursor frequency and affinity characteristics is just beginning, both for mice and humans. In comparison to the epitope-specific T cell literature [22,23], epitope-specific naive B cell data are limited. Precursor frequencies for several proteins have been determined, but not for individual epitopes on those proteins [24,25] (Table 1). Both for protein antigens and chemical haptens, naive B cell affinity data are available for a limited number of epitopes. Epitope-specific naive B cell frequencies have been determined for several small chemical haptens, including 4-hydroxy-3-nitrophenyl-acetyl (NP) (Table 1). In most popular hapten and protein B cell response animal models, cognate naive B cells are common. For example, the widely studied hapten NP has a precursor frequency of ∼1 in 5000 B cells in mice.

Available experimental mouse models for epitope-specific B cell responses have primarily used the hapten moiety NP, which may not be a good reflection of the B cell and T cell biology challenges facing epitope-specific B cell responses to pathogens. This may be particularly true for HIV bnAb responses, as bnAb development requires extensive affinity maturation, whereas the focus of NP affinity maturation is largely on a single amino acid change; additionally, the precursor frequencies of naive B cells that can recognize HIV bnAb epitopes is low [7]. Direct immunization of BCR or V-gene knock-in mice provided the first encouraging *in vivo* evidence that eOD-GT antigens can prime naive VRC01-class B cells [15,16,18,20], but the precursor frequencies in such mice are supraphysiological and the affinities may be supraphysiological as well. Therefore, we developed a mouse B cell transfer experimental model to precisely control precursor frequencies of naive VRC01-class B cells expressing the VRC01 inferred-germline HC and LC (VRC01^gHL^) to assess the roles of B cell precursor frequencies and affinities on immune responses [8]. B cell precursor frequency and BCR affinity were observed to be major factors in B cell competition and immunodominance. Interdependent roles of precursor frequency, antigen affinity, and antigen avidity determined the competitive success of B cells in GC following immunization. When the epitope-specific naive B cell precursor frequency and antigen affinity were matched to the human physiological range (1 in 10^6^ B cells and 0.5 μm K_D_ by SPR), VRC01-class B cells were robustly recruited and were able to effectively compete in GCs, dominating ∼90% of GCs containing VRC01-class B cells, with substantial affinity maturation [8]. Under lower affinity conditions the B cells were less competitive (14 μm *K*_D_ by SPR). Equally important, we found that VRC01-class B cells formed memory B cells under these physiological affinity and precursor frequency conditions. While human clinical trials are the critical test, these mouse model data provide encouraging evidence that VRC01-class naive B cells are sufficiently common in humans and eOD-GT8 has sufficient affinity for those cells that eOD-GT8 60-mers have a reasonable chance of success in the first-in-class germline-targeting vaccine human clinical trial.

A later study corroborated that B cell precursor frequency and affinity have substantial effects on GC responses to a protein antigen, utilizing a synthetic intermediate VRC01-class BCR knock-in mouse as a donor (3BNC60^SI^) with Env trimer CD4bs immunogens of varying affinity [26]. While the studies used different mice, antigens, and epitope valencies, the overall results from both studies appear highly consistent. Comparing the two studies, the 14 μm Surface Plasmon Resonance (SPR) affinity interaction in [8] likely approximates the 40 μm Bio-Layer Interferometry (BLI) affinity in [26], as CD4bs affinities measured by SPR were found to be twofold to threefold tighter on average than affinities measured by BLI [21]. When precursor frequencies were in the low physiological range (1 in 10^6^ B cells, approximately 60 cells engrafted per mouse) and antigen affinity was within a moderate range (14 μm SPR, 40 μm BLI) modest VRC01-class B cell responses were observed in both studies. These data suggest that low affinity epitope-specific B cells are only likely to be a substantial fraction of GC responses when starting from high precursor frequencies.

A question related to precursor frequency is: How many human B cells encounter the antigen from an immunization? This impacts the inferred physiological relevance of rare precursor frequencies. A reasonable estimate can be extrapolated from known numbers. An average human LN has ∼50 million lymphocytes, and ∼25 million B cells. LNs are frequently found in clusters, thus it is plausible that 2–4 LNs in a drainage region will uptake antigen (∼75 million B cells). If the immunization is strong, it appears to engage the primary LN ‘station’ and a secondary station (the first lymphatic drainage site and the second closest drainage site) (unpublished data); that would double the number again (150 million B cells). If the immunizations were done bilaterally, that number would double again (300 million B cells). B cells exhibit a resting LN dwell time of approximately 10–16 hours [27, 28, 29]; in addition, it is reasonable to assume that naive B cells can be readily recruited into the immune response during at least the first 48 hours [30]. LNs can swell after infection or immunization, filling with more B cells [31]; although access of new cells to inflamed LNs can become limited over time [32]. One model predicts a fourfold increase in accumulation of cells in stimulated versus unstimulated LNs [29]. There is uncertainty regarding the timing and combination of these factors; but, roughly estimated, recirculation and swelling factors may increase the number of B cells encountering the immunogen fourfold, to 1.2 × 10^9^. Therefore, it is not unreasonable to estimate that 1.2 × 10^9^ B cells are potentially ‘screened’ *in vivo* for participation in a local immune response in an immunized person. Future studies to assess the number of B cells encountering antigen post-immunization would be of value for human vaccine immunology.

## Subclasses of epitope-specific naive B cells

The hope is that some VRC01-class naive B cells would be capable of affinity maturing into VRC01-class bnAbs over time upon immunization with additional immunogens. Epitope-specific naive B cells can be further categorized into Ab subclasses based on BCR sequence characteristics [21]. The characteristics of each subclass and frequency of that subclass within the repertoire could substantially impact the relevance of that naive B cell subclass for vaccine development. For example, one critical developmental aspect of the VRC01 bnAb was a somatic deletion in the L-CDR1; however, that potential requirement is bnAb subclass conditional [33]. N6-subclass, VRC23-subclass, and PCIN63-subclass bnAbs (using LC VK1-33, VK3-15, and VK1-5, respectively) do not require deletion events. These subclasses in the repertoire can each be conceptualized as independent departure points, or beachheads, for starting HIV CD4bs bnAb lineages via immunization, each with positive and negative characteristics. Additionally, specific paratope and sequence properties shared by VRC01-class bnAbs, inferred precursors of VRC01-class bnAbs, and VRC01-class naive B cells can be defined and categorized [21]. It is reasonable to consider that the more VRC01-class bnAb sequence features shared by a given VRC01-class naive B cell, the more likely it is that the naive B cell has the potential to develop into a VRC01-class bnAb in an appropriate immunization regimen.

**Figure 2:**
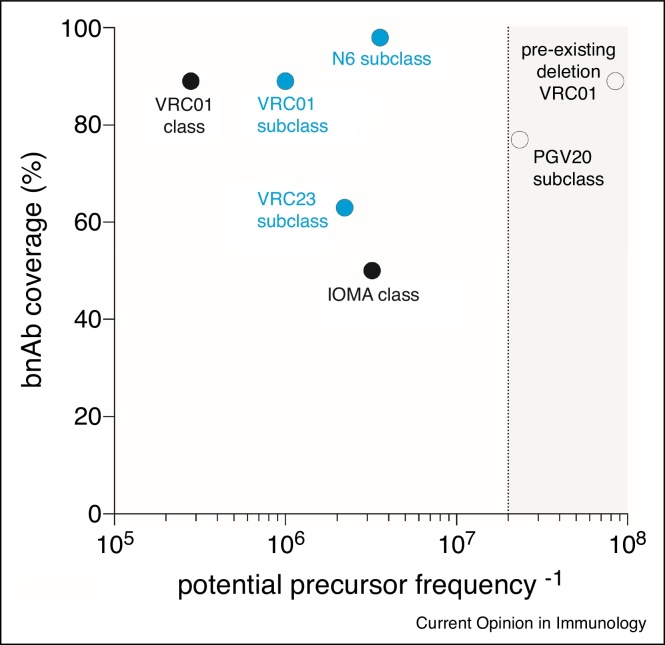
Consideration of B cell frequencies and bnAb coverage. ‘Potential bnAb precursor frequency’ is plotted as the inverse of naive B cell frequency (i.e. 1 in ‘*x*’ number of B cells) for each class or subclass. The IOMA class naive B cell frequency may be underestimated, as eOD-GT8 was not specifically designed to target IOMA-like cells. However, based on human B cell repertoire bioinformatics, the frequency of the IOMA lambda LC is expected to be lower than or similar to that of the kappa VRC01-class LCs, as lambda LCs are longer and less common than kappa LCs. BnAb class and subclass percentage neutralization coverage are plotted using values of the named bnAb (i.e. ‘VRC01 class’ coverage value is the neutralization of VRC01 bnAb [47]). Detected subclasses are shown in blue [21]. Naive B cell subclasses that are below the limit of detection are plotted in the grayed area (plotted values vary due to differences in experimental approach).

Another important factor to consider is the neutralization breadth of the bnAb being targeted for generation. An analysis of the precursor frequency of naive B cells with bnAb potential compared with the bnAb neutralization breadth of worldwide HIV stains provides a means to prioritize target subclasses and help interpret human vaccine trial results (Figure 2). For example, VRC01-class B cells may be preferred as targets compared to IOMA-class (another CD4bs-specific bnAb [34]) because VRC01-class naive B cells appear to be more frequent in the human repertoire and VRC01-class bnAbs have greater neutralizing coverage. As a second example, among VRC01-subclasses the N6-subclass naive B cells are comparably frequent in the repertoire with other subclasses, while the N6 bnAb neutralization coverage is near complete [35]. In contrast, the PGV20 bnAb has good neutralization coverage, but PGV20-class naive B cells are below the limit of detection in the human repertoire and are thus likely a difficult vaccine design target. Similarly, many VRC01-class bnAbs depend on BCR CDR deletions/insertions for bnAb activity, yet naive VRC01-class human B cells with such an attribute have not been identified (frequency less than 1 in 100 million B cells). Overall, both the frequency of naive B cell precursors and neutralizing coverage are valuable factors to consider when targeting B cells for bnAb generation by immunization.

## Technical considerations

A combination of fluorescently labeled antigen probes and flow cytometry can be used to identify antigen-specific B cells. This method has been a boon to the isolation of affinity-matured B cells expressing BCRs with high affinity for antigen to myriad antigens [36,37]. However, naive B cells have not undergone affinity maturation or clonal expansion. Therefore, identifying and isolating rare, low affinity antigen-specific naive B cells as described above requires additional considerations. First, a large number of naive B cells must be screened. Antigen-specific naive B cells have not undergone antigen-induced expansion and are therefore much rarer than antigen-experienced B cells. It is not uncommon to screen 1 × 10^9^ PBMCs from a single donor in the search for antigen-specific naive B cells in humans. Second, fluorescently labeled antigen probes must be carefully chosen. These probes are generally comprised of an antigen of interest, a fluorochrome, and a conjugation/multimerization linker (such as biotin + streptavidin). The conformation of the protein antigen probe should be a faithful mimic of the immunogen or viral protein of interest. This was a major problem for HIV Env trimer for decades, as well as a major problem for understanding B cells with neutralizing Ab specificities against RSV. Probes may also detect B cells that are not of interest, including B cells specific for the fluorochrome, or the conjugation/multimerization domain. The use of two ‘positive’ antigen probes labeled with differing fluorescent molecules can greatly enhance identification of B cells specific for the antigen of interest. Fluorochromes should be selected carefully, considering brightness (stain index), compensation (spectral overlap), and repeated fluorochromes in tandem dyes (use of PE and PE-Cy7 fluorochromes on positive probes would be expected to isolate PE-specific B cells). In addition, low molecular weight fluorochromes, such as Alexa Fluors, likely provide fewer potential epitopes for B cell recognition than large protein molecules, such as PE. Varying the method of fluorescent labeling of the antigen (i.e. biotin + streptavidin versus direct amine labeling) can avoid isolating unwanted B cells specific for the linker. Protein expression tags used for purification, such as His tags, are another potential source of probe specific, but not antigen-specific naive B cells, as His tags are highly immunogenic *in vivo* [38]. Third, the use of a ‘negative’ probe presenting a mutated epitope can provide two major benefits. It increases overall specificity by avoiding non-specific ‘sticky’ B cells which bind all probes. An alternative ‘negative’ probe approach uses Alexa647 conjugated to SA-PE plus biotin (biotin-PE*AF647), to identify and gate out B cells specific for non-antigen probe components [39]. In addition, epitope ‘knock out’ negative probes allow for the identification of B cells specific for the epitope of interest on the antigen [19,21]. This is particularly informative when assessing the repertoire of B cells specific for antigens designed to engage a particular B cell specificity, such as germline-targeting immunogens.

Careful flow cytometry sorting can still be insufficient for distinguishing rare, low affinity antigen-specific naive B cells from background noise, and successful antigen-specific naive B cell isolation can be strengthened by combining an initial flow cytometry screen with a downstream B cell culture antigen-specificity validation step. One such approach is to grow single cell cultures from sorted naive B cells of interest, stimulating the B cells to secrete IgM. The culture supernatants can then be screened for antigen-specificity by ELISA. In this way, many different antigens can also be tested to aid fine epitope determination and/or assess polyreactivity. For a cell to be considered antigen-specific at the end of this multi-assay screening process, multiple specificity criteria are required to be achieved. Together, the flow cytometry and B cell culture strategies form a stringent two-stage, multiple-validation methodology to isolate rare, low affinity antigen-specific naive B cells [17,21].

## Summary

It is worthwhile to examine the antigen-specific naive B cell repertoire for engineered protein candidate vaccines. The technology is available to do so. Studying the naive B cell repertoire in the context of vaccine candidates allows for iterative vaccine design based on the composition of the naive B cell repertoire and the immunogen binding characteristics of those naive B cells. Probing the naive human B cell repertoire also provides knowledge useful for ranking or triaging candidate vaccine immunogens. Repertoire studies may reveal unpredicted immunodominant off-target specificities, which can then be engineered out of the candidate vaccine before costly human clinical trials. The knowledge gained also allows for a better understanding of B cell biology, including B cell competition and immunodominance. Greater study of this research area is warranted.

## Conflict of interest statement

IAVI and the Scripps Research Institute have filed a patent relating to the eOD-GT8 immunogens in this manuscript, which included inventor W.R.S. W.R.S. is a cofounder and stockholder in CompuVax Inc., which has programs in non-HIV vaccine design that might benefit indirectly from this research.
